# Experiences of hospital care for people with multiple long-term conditions: a scoping review of qualitative research

**DOI:** 10.1186/s12916-023-03220-y

**Published:** 2024-01-17

**Authors:** Sue Bellass, Thomas Scharf, Linda Errington, Kelly Bowden Davies, Sian Robinson, Adam Runacres, Jodi Ventre, Miles D. Witham, Avan A. Sayer, Rachel Cooper

**Affiliations:** 1 Department of Sport and Exercise Sciences, Manchester Metropolitan University, Manchester, UK; 2 Population Health Sciences Institute, Faculty of Medical Sciences, Newcastle University, Newcastle Upon Tyne, UK; 3 School of Biomedical Nutritional and Sport Sciences, Newcastle University, Newcastle Upon Tyne, UK; 4 AGE Research Group, Translational and Clinical Research Institute, Faculty of Medical Sciences, Newcastle University, Newcastle Upon Tyne, UK; 5 NIHR Newcastle Biomedical Research Centre, Newcastle University, Newcastle Upon Tyne NHS Foundation Trust and Cumbria, Northumberland, Tyne and Wear NHS Foundation Trust, Newcastle Upon Tyne, UK; 6 NIHR ARC Greater Manchester, School of Health Sciences, Faculty of Biology, Medicine and Health, University of Manchester, Manchester, UK

**Keywords:** Multiple long-term conditions, Multimorbidity, Qualitative research, Hospital care, Lived experience

## Abstract

**Background:**

Multiple long-term conditions—the co-existence of two or more chronic health conditions in an individual—present an increasing challenge to populations and healthcare systems worldwide. This challenge is keenly felt in hospital settings where care is oriented around specialist provision for single conditions. The aim of this scoping review was to identify and summarise published qualitative research on the experiences of hospital care for people living with multiple long-term conditions, their informal caregivers and healthcare professionals.

**Methods:**

We undertook a scoping review, following established guidelines, of primary qualitative research on experiences of hospital care for people living with multiple long-term conditions published in peer-reviewed journals between Jan 2010 and June 2022. We conducted systematic electronic searches of MEDLINE, CINAHL, PsycInfo, Proquest Social Science Premium, Web of Science, Scopus and Embase, supplemented by citation tracking. Studies were selected for inclusion by two reviewers using an independent screening process. Data extraction included study populations, study design, findings and author conclusions. We took a narrative approach to reporting the findings.

**Results:**

Of 8002 titles and abstracts screened, 54 papers reporting findings from 41 studies conducted in 14 countries were identified as eligible for inclusion. The perspectives of people living with multiple long-term conditions (21 studies), informal caregivers (*n* = 13) and healthcare professionals (*n* = 27) were represented, with 15 studies reporting experiences of more than one group. Findings included poor service integration and lack of person-centred care, limited confidence of healthcare professionals to treat conditions outside of their specialty, and time pressures leading to hurried care transitions. Few studies explored inequities in experiences of hospital care.

**Conclusions:**

Qualitative research evidence on the experiences of hospital care for multiple long-term conditions illuminates a tension between the desire to provide and receive person-centred care and time pressures inherent within a target-driven system focussed on increasing specialisation, reduced inpatient provision and accelerated journeys through the care system. A move towards more integrated models of care may enable the needs of people living with multiple long-term conditions to be better met. Future research should address how social circumstances shape experiences of care.

**Supplementary Information:**

The online version contains supplementary material available at 10.1186/s12916-023-03220-y.

## Background

Multiple long-term conditions (MLTC)—the co-existence of two or more long-term conditions in an individual—are becoming more common, with far-reaching consequences for populations and health services worldwide [1–3]. Although the definition and operationalisation of the concept of MLTC is highly variable [4] and a need for greater consistency has led to recent efforts to reach a consensus [5], the term MLTC is generally understood to be the experience of at least two long-term health conditions of long duration, including non-communicable diseases, infectious diseases and mental health conditions [2].

Inconsistencies in the definition and characterisation of MLTC have led to major variations in prevalence estimates [4, 6]; however, it is estimated that one in four of UK adults live with MLTC [7, 8] and that prevalence is increasing. The proportion of British adults aged over 65 years with MLTC is predicted to rise from 54% in 2015 to 68% by 2035 [9]. Multi-country studies suggest similarly high prevalence of MLTC in other high-income settings, with MLTC in low- and middle-income countries advancing towards equivalent levels [10, 11]. The accumulating evidence of the current and anticipated scale of MLTC, and their impact on quality of life and demand for healthcare, have led to calls to prioritise MLTC research [2].

People living with MLTC are more likely to experience lower quality of life, lower healthy life expectancy and poorer health outcomes than people with no or a single long-term condition [12, 13], and there is a growing awareness that clinical education, evidence-based guidelines and health services, typically oriented around single conditions, are fundamentally unsuited to the needs of this population [14–16]. This can be keenly observed in secondary and tertiary care, which, in recent years, have been characterised by greater specialisation [17]. While improving care and outcomes for single conditions, an increasing focus on specialised care may hinder the development of coordinated care able to address co-existing conditions in people with MLTC.

Recognising the need to better understand hospital care for MLTC, there is an important role for studies that can elucidate the lived experiences of receiving or delivering care. We therefore chose to focus in this review on qualitative research which, while encompassing a wide variety of methodological approaches and traditions, is characterised by the aim of producing a rich understanding of the ways in which people perceive and interpret social phenomena [18, 19]. Existing systematic and scoping reviews have captured aspects of the experience of MLTC care from the perspectives of general practitioners [20] informal caregivers [21] and patients [22], while others have focussed on specific aspects of care such as coordination and integration [23, 24]. However, no reviews known to the authors specifically explore and present the experiences of hospital care delivery and receipt from the perspectives of people living with MLTC, informal caregivers and healthcare professionals. To address this important gap, we undertook a scoping review to identify the breadth of relevant literature, describe the key concepts explored and highlight gaps in the knowledge base [25, 26].

In line with the core objectives of scoping reviews [27], we specifically aimed to address the following three research questions:

1. What is the nature, range and extent of published qualitative literature exploring hospital care experiences of people living with MLTC, informal caregivers and healthcare professionals?
2. What experiences of hospital care have been reported in the literature?
3. What gaps exist in the knowledge base that might be addressed by future research?

## Methods

Our approach to the review was informed by Arksey and O’Malley’s scoping review framework [25] and recently updated guidance on scoping review methodology [26]. In line with these frameworks and guidelines, a protocol was created which pre-specified the inclusion and exclusion criteria for the review (see Additional file 1) [4, 25, 28–34]. This scoping review was reported according to the Preferred Reporting Items for Systematic Reviews and Meta-Analyses extension for Scoping Reviews (PRISMA-ScR) [34].

### Searching for relevant studies

Seven databases were searched systematically to identify eligible studies. The search strategies were formulated and executed by a medical librarian (LE) using the PICoS (Population-Phenomenon of Interest-Context- Study type) framework (see Table 1).

**Table 1:** Population-Phenomenon of Interest-Context-Study (PICoS) framework

P	Population	People with experience of multiple long-term conditions (MLTC; including both physical and mental health conditions) as patients, family members and friends who provide support, or staff delivering care
I	Phenomenon of Interest	Experience
Co	Context	Hospital care
S	Study type	Qualitative

The search strategy involved combining both subject index and keyword terms covering the following concepts: MLTC, secondary care and qualitative research. Full details of the search strategies can be found in Additional file 2. The following databases were independently searched from 1st Jan 2010 to 22nd June 2022: Medline, Embase and PsycINFO (via OVID), Web of Science, Scopus, CINAHL via EBSCO, and Social Science Premium via Proquest. We opted to restrict the date to 2010 onwards as, given regular restructuring of hospital services and the increasing prevalence of MLTC, we wished to identify studies that reflected experiences most likely to be relevant to the current context of hospital care. Study titles and abstracts were uploaded to systematic review management software (Covidence) where they were deduplicated and screened against inclusion and exclusion criteria (see Table 2). Consistent with scoping review methodology [26], published studies were not excluded from the review on the basis of poor methodological quality.

**Table 2:** Inclusion and exclusion criteria

Inclusion criteria	Exclusion criteria
Primary qualitative studies (or mixed methods studies with a qualitative component) that report evidence relating to hospital care for people living with MLTC, informal caregivers, or care professionals	Quantitative research studies, intervention studies, study protocols, conference abstracts, or literature reviews
Studies published from 1st Jan 2010	Studies with a focus on everyday life or self-management of MLTC
English language	Studies where study participants are recruited on the basis of older age rather than MLTC
	Studies undertaken exclusively in primary care settings

Screening of titles and abstracts was carried out independently by six members of the review team (SB, LE, KBD, AR, JV, RC) with two reviewers screening each record. Any uncertainty or disagreement about inclusion was resolved through in-depth discussion between SB and RC. The reference lists of eligible studies and results from forward citation tracking were screened to identify additional articles.

Once eligible studies had been identified, a data extraction chart (see Additional file 3) was created following discussions among three authors (SB, RC, TS) and populated by one author (SB). Extracted data included author(s), year of publication, journal, definition of MLTC, theoretical framework, aims, methods, setting, health conditions, participants, findings, and author conclusions. In line with established methodological guidance from Arksey and O’Malley [25] and Peters et al. [26], we did not conduct formal critical appraisal of the included studies.

### Collating, summarising and reporting results

Using the data extracted, studies were categorised according to country of origin, diagnosis-specific and non-diagnosis-specific research and perspectives of participants (people living with MLTC, informal caregivers and healthcare professionals). Consistent with recent guidance on scoping review methodology [26], the results are summarised narratively rather than analysed thematically.

## Results

### Overview of studies

**Fig. 1:**
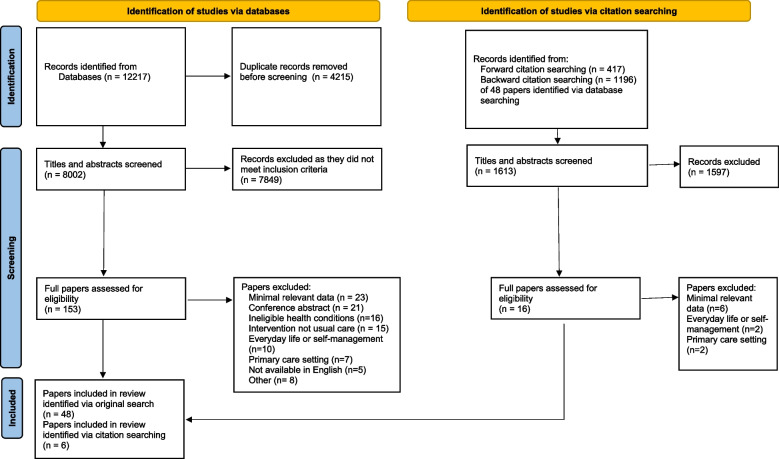
PRISMA flow diagram

We screened 8002 records from electronic database searches and a further 1613 records identified through citation tracking (see Fig. 1). A total of 54 papers [35–88] met the inclusion criteria for the review and these reported on findings from 41 unique studies (see Table 3 for a summary of these papers).

**Table 3:** Key features of included papers ordered alphabetically and chronologically within studies

Lead author, year and reference number	Country	Aim	Study design and data collection	Setting	Participant group	Number of participants	Health condition	Age range (years) of people living with MLTC (where applicable)
Aebi, 2021 [35]	Switzerland	To investigate mental-somatic multimorbidity in hospital settings.	Generic qualitative Cross-sectional Interviews	Three general hospitals	HCPs	18	Not diagnosis specific	
**2 papers based on one study**								
Andersen, 2018 [36]	Denmark	Not explicitly stated, but to explore professional collaboration regarding patient pathways from EMU for elderly people with multiple chronic illnesses	EthnographyTime period not stated Observation	Cross-sectoral: regional (secondary care emergency units), municipalities (community care, public health), primary care	Patients; HCPs	10 patients; unclear how many HCPs were observed	Not diagnosis specific	68–89 (mean = 78.5)
Andersen, 2019 [37]		To explore intersectoral collaboration and the creation of patient pathways for elderly people (65 +) with multiple chronic conditions, from emergency unit to home (or other care facility)						
Backman, 2018 [38]	Canada	To explore experiences of transitions across health care settings.	Participatory visual narrative Cross-sectional Photo walkabout interviews		Patients, informal caregivers	9	Not diagnosis specific	56–94
Bartlett, 2012 [39]	UK	To understand: how healthcare professionals assess the needs of an older person dying from cancer with a coincidental dementia, whether older people with cancer and dementia have differing care needs from those who do not have dementia, how healthcare professionals communicate with an older person dying from cancer with a coincidental dementia	Guided by Colaizzi’s phenomenological method Cross-sectional Interviews	Acute hospital	HCPs and a chaplain	5	Cancer and dementia	
Ben-Menahem, 2021 [40]	Switzerland	To understand and develop a framework for care providers’ perceptions of complexity	Phenomenology / IPA Cross-sectional Interviews	HIV outpatient care	HCPs	31	HIV and other morbidities	
**3 papers based on one study**								
Bosire, 2021 [41]	South Africa	To explore patients’ experiences of accessing healthcare for comorbid HIV/AIDS and diabetes	EthnographyApr 2018–Jun 2019 ObservationInterviews	Public tertiary hospital	Patients	15	HIV and diabetes	40–70
Bosire, 2021 [42]		To explore how the health system functions to care for patients with comorbid type 2 diabetes and HIV/AIDS at a tertiary hospital			HCPs	30		
Bosire, 2021 [43]		To explore provider perspectives on person-centred care for people with HIV and comorbid diabetes			HCPs	30		
Bunn, 2017 [44]	UK	To explore the impact of dementia on access to non-dementia services and identify ways of improving service delivery for this population	Generic qualitative Cross-sectional Interviews and focus groups	Primary and secondary care	Patients, Informal caregivers, HCPs	117 (28 patients, 33 informal caregivers, 56 HCPs)	Dementia and other morbidities	59–94 (median = 82.5)
Carusone, 2017 [45]	Canada	To explore the obstacles and challenges faced by complex patients during hospital discharge and post-discharge transition	Case study Repeated interviews around 6 weeks	13-bed subacute hospital	Patients	9	HIV and other morbidities	23–54
Cho, 2021 [46]	USA	To assess HCPs’ expectations of diabetes management during cancer treatment and to identify possible communication barriers between primary and secondary care	Grounded theory Cross-sectional Interviews	Three hospitals	HCPs	10 oncologists	Cancer and type 2 diabetes	
Cook, 2018 [47]	Australia	To examine how HCPs working in a cancer service undertake treatment decision-making and recommendations and how this is affected by medical and social judgements	Generic qualitative Cross-sectional Interviews	Large cancer care service	HCPs	9	Cancer and dementia	
Cullinan, 2015 [48]	Ireland	To identify hospital doctors’ perceptions as to why potentially inappropriate prescribing (PIP) occurs, to identify barriers to addressing PIP and to determine which intervention types would improve prescribing	Generic qualitative Cross-sectional Interviews	Public and voluntary hospitals	HCPs	22	Not diagnosis specific	
Doos, 2015 [49]	UK	To identify issues regarding management of type 2 diabetes in patients with cancer	Grounded theory Cross-sectional Interviews	Two cardiology and respiratory wards	Patients, Informal caregivers	11 (6 patients, 5 caregivers)	Heart failure and Chronic Obstructive Pulmonary Disease (COPD)	Patients: 62–91
Duthie, 2017 [50]	Canada	To explore cancer patients’ experience with multimodal treatments, complex healthcare needs and navigating the healthcare system	Generic qualitative Cross-sectional Interviews	Cancer centre in university hospital	Patients	10	Cancer and other morbidities	52–79
Ekdahl, 2012 [51]	Sweden	To explore physicians’ perceptions and experiences of including elderly patients with multimorbidity in clinical decision-making	Grounded theory Cross-sectional Focus groups	Three hospitals in two counties	HCPs	30	Not diagnosis specific	
Fabricius, 2021 [52]	Denmark	To explore the determinants of patient involvement in decisions made in the ED about the patient’s medication	Ethnography 5 months Interviews Observations	Two medical emergency departments in a university hospital	HCPs	48 (observation) 20 (interviews}	Not diagnosis specific	
Gallagher, 2015 [53]	Australia	To identify which older people emergency nurses perceive as using more nursing resources	Generic qualitative Cross-sectional Focus groups	Tertiary referral university hospital emergency department	HCPs	27	Not diagnosis specific	
Goebel, 2016 [54]	USA	To identify issues regarding management of type 2 diabetes in patients with cancer	Generic qualitative Cross-sectional Focus groups	Two outpatient cancer centres	Patients, HCPs	25 (5 patients, 20 HCPs)	Cancer and diabetes	Patients: mean = 59.4
**5 papers based on one study**								
Griffiths, 2020 [55]	UK	To explore cancer treatment decision-making in comorbid cancer and dementia	Ethnography Sep 2018 to May 2019 46 h of participant observations 9 h of non-participant observations 37 interviews Medical notes review Informal conversations	Two English Trusts which provide local cancer services and more specialist regional provision	Patients; Healthcare professionals (HCPs); Informal caregivers	58 (17 patients, 22 relatives, 19 staff)	Cancer and dementia	45–88 (mean = 75)
Surr, 2020 [56]		To explore the role of supportive networks in assisting and enabling people with comorbid cancer and dementia to receive hospital-based cancer treatment and care						
Ashley, 2021 [57]		To examine the hospital-based cancer care and treatment challenges and support needs of people with dementia						
Griffiths, 2021 [58]		To understand how oncology services balance the needs of patients who have cancer and dementia						
Surr, 2021 [59]		To explore the challenges of navigating cancer treatment and care for people with comorbid cancer and dementia, their family members and oncology staff						
Hansson, 2018 [60]	Sweden	To describe the experiences of healthcare professionals of the obstacles and opportunities for collaboration with patients and their relatives and between providers of care	Generic qualitative Cross-sectional Focus groups	Hospital in Sweden and affiliated community and primary care facilities	HCPs	24	Not diagnosis specific	
Hultsjö, 2013 [61]	Sweden	To explore mental healthcare staff’s experiences of diabetes care given to people with psychosis	Generic qualitative Cross-sectional Interviews	Psychiatric outpatients	HCPs	12	Psychosis and diabetes	
Huque, 2020 [62]	Bangladesh	To explore the experiences of people living with comorbid depression and tuberculosis of hospital care	Generic qualitative Cross-sectional Interviews	119-bed chest hospital	Patients, Informal caregivers, HCPs	23 (12 patients, 4 informal caregivers, 4 HCPs, 3 policymakers)	TB and depression	18–51 +
Jayakody, 2021 [63]	Australia	To explore the experiences and perceptions of unplanned hospital readmissions from the perspective of Aboriginal and Torres Strait Islander people with multiple chronic disease	Generic qualitative Cross-sectional Interviews	Two tertiary hospitals	Patients	15	Multiple chronic diseases including CVD, chronic respiratory disease, diabetes, cancer, renal disease, osteoporosis, mental health conditions	37–83 (median 68)
**3 papers based on one study**								
Kuluski, 2013 [64]	Canada	To investigate what is important in care delivery from the perspective of hospital inpatients with complex chronic disease	Generic qualitative Cross-sectional Interviews Open-ended question data	Continuing care hospital	Patients	116 total (not all contributed to each aspect of the study)	Range of health conditions, most common were musculoskeletal conditions followed by stroke and multiple sclerosis	< 44 (*n* = 13; 12%) 45–64 (*n* = 52; 47%) 65 + (*n* = 46; 41%) < = 44 to 65 +
Ho, 2015 [65]		To better understand the discharge experience of people with multiple chronic diseases						
Kuluski, 2015 [66]		To explore factors that may serve as tipping points into poor health from the perspective of hospitalised patients with multimorbidity						
**2 papers based on one study**								
Kumlin, 2020 [67]	Norway	To explore how elderly patients with complex health problems engage in and interact with their care trajectory across different healthcare systems	Generic qualitative Cross-sectional Interviews	One rural hospital, one urban hospital, six municipalities	Patients HCPs	11 25	Not diagnosis specific	65–91
Kumlin, 2021 [68]		To uncover the work that HCPs undertake to achieve coherent and comprehensive care for elderly patients with multiple health problems						
Lekas, 2012 [69]	USA	To examine the reasons underlying the low rate of HCV treatment among HIV + patients	Generic qualitative Cross-sectional Interviews	Two urban hospitals	HCPs	17	HIV/ HCV (hepatitis C virus	
Lilleheie, 2020 [70]	Norway	To explore older patients’ subjective experiences of quality of health services in and after hospital	Phenomenology/ IPA Repeated interviews (n = 2) during and 30 days after hospitalisation	Acute geriatric ward	Patients	22 (18 retained in the study)	Not diagnosis specific	82–100 (mean = 92)
**2 papers based on one study**								
Lo, 2016 [71]	Australia	To explore the perspectives of patients and carers on factors influencing healthcare of people with comorbid diabetes and CKD	Generic qualitative Cross-sectional Interviews & focus groups	Four tertiary health services in two large Australian cities	Patients and informal caregivers HCPs	58 patients 8 informal caregivers 65	Diabetes and CKD	41–90 (majority aged 61–70)
Lo, 2016 [72]		To explore the perspectives of general practitioners and tertiary health-care professionals concerning key factors influencing health-care of diabetes and CKD						
Malley, 2018 [73]	USA	To describe the preoperative care transitions experience of older adults with multiple chronic conditions and their relatives and to examine preoperative engagement and their reflections postoperatively	Generic qualitative Repeated interviews (2 in around 4 weeks)	975-bed medical centre	Patients, informal caregivers	16 (11 patients, 5 relatives	Not diagnosis specific	median = 81
Martin, 2022 [74]	UK	To explore the role of family caregivers in making cancer treatment decisions for older women with pre-existing dementia and breast cancer, particularly the decision between surgery and non-surgical treatment	Generic qualitative Cross-sectional Interviews	13 breast cancer services	Informal caregivers	8	Dementia and breast cancer	
Mason, 2016 [75]	UK	To report the experiences and perceptions of people with advanced multimorbidity to inform improvements in palliative and end-of-life care	Generic qualitative Repeated interviews over 5–9 months	Acute admissions unit in Scotland, English general practice, respiratory outpatient clinic	Patients, informal caregivers	87 interviews (42 patient alone, 2 informal caregivers alone, 43 patient-caregiver dyad)	Not diagnosis specific	55–92 average = 76
**2 papers based on one study**								
McWilliams, 2018 [76]	UK	To explore cancer-related information needs and decision-making experiences of patients with cancer and comorbid dementia, their caregivers and oncology HCPs	Generic qualitative Cross-sectional Interviews	Regional tertiary care cancer centre	Patients; HCPs; Informal caregivers Patients; Informal caregivers	31 (10 patients, 9 informal caregivers, 12 HCPs) 19 (10 patients, 9 informal caregivers)	Cancer and dementia	39–93
McWilliams, 2020 [77]		To explore decision-making and treatment options for people who live with dementia and cancer						
Mikkelsen, 2020 [78]	Denmark	To describe psychiatric nurses’ and diabetes nurses’ experiences of care with hospitalised patients with schizophrenia and diabetes	Phenomenology/ IPA Cross-sectional Interviews	Endocrinology ward and psychiatric ward	HCPs	8	Schizophrenia and diabetes	
Neiterman, 2015 [79]	Canada	To examine how patients (with multiple chronic health conditions) experience transitions to community from hospitals	Generic qualitative Cross-sectional Interviews		Patients Informal caregivers	36 (17 patients, 19 informal caregivers)	Range of conditions	70–89 (average 79)
**2 papers based on one study**								
Nikbakht Nasrabadi, 2021 [80]	Iran	To explore nurses’ experiences of transitional care in multiple chronic conditions	Generic qualitative Cross-sectional Interviews	University hospitals in two large cities	HCPs	15	Diabetes and other morbidities	
Nikbakht Nasrabadi, 2021 [81]		To explore family caregivers’ experiences of transitional care in diabetes with concurrent chronic conditions			Informal caregivers	15		
Perrault-Sequeira, 2021 [82]	Canada	To identify and explore the networks of care providers in a sample of hospitalised complex patients and better understand the nature of their attachment to these providers	Grounded theory Cross-sectional Interviews		Patients	30	Not diagnosis specific	Mean age 69.5
Rivers, 2020 [83]	UK	To understand the mindset of doctors and pharmacists as they embark upon prescribing in a multimorbidity and polypharmacy context during routine practice at a hospital acute admissions unit and to evaluate to what extent attitudes relate to existing theory and models of prescribing decisions	Phenomenologically oriented Cross-sectional Focus groups		HCPs	48	Not diagnosis specific	
Schiøtz, 2017 [84]	Denmark	To investigate quality of care for people with multimorbidity	Generic qualitative Cross-sectional Focus groups	University hospital	HCPs	18	Range of cardiometabolic conditions, depression and COPD	
Schonfeld, 2012 [85]	USA	To explore physicians’ experiences in conducting end-of-life conversations with elderly patients with comorbidities	Generic qualitative Cross-sectional Focus groups		HCPs	32	Not diagnosis specific	
Verhoeff, 2018 [86]	The Netherlands	To investigate patients’ experiences, beliefs and understandings of the current secondary care of patients with multiple chronic conditions	Generic qualitative Cross-sectional Interviews	Internal medicine and geriatric outpatients department	Patients	8	Not diagnosis specific	67–92 (median 71.5)
Witham, 2018 [87]	UK	To explore the experience of carers who have supported a relative with cancer and dementia using a narrative approach	Narrative Cross-sectional Interviews	Regional cancer treatment centre	Informal caregivers	7	Cancer and dementia	
Younas, 2022 [88]	Pakistan	To determine nurses’ perceived barriers to the delivery of person-centred care to complex patients with multiple chronic conditions in acute care settings	Generic qualitative Cross-sectional Interviews	Two hospitals	HCPs	19	Not diagnosis specific	

The studies were conducted in 14 countries, with the majority from the UK (9 studies, 14 papers) and Canada (6 studies, 8 papers). Four studies were conducted in low- and middle-income countries (Bangladesh, Iran, Pakistan and South Africa) with the remainder from high-income countries in Western Europe, Northern America or Australasia (Table 4). The key concepts underpinning studies were notably similar, despite the various contexts in which the studies were undertaken. Where there were clear differences between healthcare conditions, countries, or the perspectives of people with MLTC, informal caregivers or healthcare professionals, these are noted in the findings.

**Table 4:** Country of origin of included studies ordered alphabetically by number of studies and papers

Country	Number of studies	Number of papers	Reference numbers of papers
UK	9	14	[39, 44, 49, 55–59, 74–77, 83, 87]
Canada	6	8	[38, 45, 50, 64–66, 79, 82]
USA	5	5	[46, 54, 69, 73, 85]
Australia	4	5	[47, 53, 63, 71, 72]
Denmark	4	5	[36, 37, 52, 78, 84]
Sweden	3	3	[51, 60, 61]
Norway	2	3	[67, 68, 70]
Switzerland	2	2	[35, 40]
South Africa	1	3	[41–43]
Iran	1	2	[80, 81]
Bangladesh	1	1	[62]
Ireland	1	1	[48]
Pakistan	1	1	[88]
The Netherlands	1	1	[86]

Around half of the studies (*n* = 21/41, 51.2%) were not diagnosis-specific, designating participants to be eligible on the basis of living with, informally supporting or delivering care for people with MLTC, or on the basis of diagnosis with at least two of a wide range of conditions. The other papers were diagnosis-specific, recruiting on the basis of two specified conditions (e.g. cancer and dementia; psychosis and diabetes) or a specified single condition with additional morbidities (e.g. HIV and multimorbidities; diabetes and multimorbidities), the latter grouping often being described as comorbidity. These study characteristics are summarised in Table 5. The age of participants living with MLTC, where a range was reported, was between 23 and 100 years. A total of 15 studies (36.6%) explicitly used age as an orienting concept in their studies, aiming to understanding the experiences of “elderly” or “older” patients.

**Table 5:** Health conditions studied

Health conditions	Number of studies	Number of papers	Reference numbers of papers
Not diagnosis-specific, or a range of conditions stated	21	25	[35–38, 48, 51–53, 60, 63–68, 70, 73, 75, 79, 82–86, 88]
Cancer and dementia	6	11	[39, 47, 55–59, 74, 76, 77, 87]
Cancer and diabetes	2	2	[46, 54]
Human immunodeficiency virus (HIV) and other morbidities	2	2	[40, 45]
Psychosis and diabetes	2	2	[61, 78]
HIV and diabetes	1	3	[41–43]
Diabetes and chronic kidney disease	1	2	[71, 72]
Diabetes and other morbidities	1	2	[80, 81]
Cancer and other morbidities	1	1	[50]
Dementia and other morbidities	1	1	[44]
Heart failure and chronic obstructive pulmonary disease	1	1	[49]
HIV and hepatitis C virus	1	1	[69]
Tuberculosis and depression	1	1	[62]

When exploring the perspectives of a single participant group, the majority of studies focussed on healthcare professionals (*n* = 17; 41.5%) compared with seven studies exploring solely the perspectives of people living with MLTC (17.1%) and two studying only the perspectives of informal caregivers (4.9%). Five studies explored views of both people living with MLTC and their caregivers (12.2%), while four (9.8%) focussed on people with MLTC and healthcare professionals, and one on informal caregivers and healthcare professionals (2.4%). Finally, five studies (12.2%) collected data on the perspectives of all three participant groups. Data collection was predominantly solely via interviews with a single person or dyad (*n* = 26; 63.4%) with some studies using focus groups (*n* = 7; 17.1%), or a combination of data collection methods, often including observation (*n* = 6; 14.6%). Most studies had a cross-sectional design (*n* = 33; 80.5%) with longitudinal designs including ethnographies (*n* = 4; 9.8%) or repeated interviews (*n* = 4; 9.8%). Sample sizes ranged from 5 to 116 for people living with MLTC, 2 to 33 for informal caregivers, and 5 to 65 for healthcare professionals. Professions represented included nurses (*n* = 21 studies; 51.2%), medical staff (*n* = 20; 48.8%), allied health professionals (*n* = 6, 14.6%), social care staff (*n* = 3; 7.3%), pharmacists (*n* = 2; 4.9%), policy makers (*n* = 1, 2.4%), or small numbers of other staff supporting people with MLTC in hospital settings such as chaplains or transport officers (*n* = 9; 22.0%).

A wide range of qualitative approaches were employed in the studies. Although the majority used generic qualitative designs that did not appear to adhere to a particular methodology (*n* = 25; 61.0%), other approaches included ethnography (*n* = 4; 9.8%), grounded theory (*n* = 4; 9.8%), and phenomenology / interpretative phenomenological analysis (*n* = 3; 7.3%). A small number of studies (*n* = 6; 14.6%) employed other methodological techniques including narrative approaches, case studies, and analysis of open-ended questions from in-person survey interviews. One study reported two designs. Studies largely did not state any underpinning theoretical framework; for those that did, the Theoretical Domains Framework (*n* = 2; 4.8%), socio-ecological framework (*n* = 2; 4.8%), and Health Outcomes Model (*n* = 1; 2.4%) guided the work. Similarly, few studies made reference to an underpinning philosophical or social theoretical stance; those that did cited phenomenology (*n* = 3, 7.3%), symbolic interactionism (*n* = 2, 4.8%), interpretivism (*n* = 2, 4.8%), pragmatism (*n* = 1; 2.4%), or Bourdieusian theory (*n* = 1; 2.4%).

### Approaches to defining MLTC

The diagnosis-specific studies described the particular conditions they were exploring. Among the studies that were not oriented around specific diagnoses or that stated a range of conditions (*n* = 21), the most common definition of MLTC given was two or more chronic conditions in an individual (*n* = 9, 42.9%). One study used the definition of one or more chronic illness [64], although recruited participants with multiple conditions. Eleven studies involving non-diagnosis-specific populations (52.4%) did not enumerate health conditions, referring instead to polypharmacy, patient complexity, or making broad reference to multiple chronic conditions. Four studies (9.8%) provided a list of conditions in their participant inclusion criteria.

A few research teams qualified their definition of MLTC, referring to the duration of the conditions [65], the need for medical management [65, 82], the lack of prioritisation of one condition over another [71], or the effect of MLTC on the person’s capabilities to carry out activities of daily living [64, 65, 67]. Three teams of authors highlighted the importance of the social context in which MLTC can occur [40, 41, 88], with one noting that defining MLTC in terms of medical complexity may obscure the influence of socioeconomic and sociocultural influences on the experience of MLTC [41].

### Coordinating service delivery

Experiences relating to service coordination and care delivery formed the main element of the findings of this review. Of all studies in the review, thirty-one (75.6%) reported findings relating to processes of interprofessional communication and service integration in specialist care settings. Interestingly, findings from all three participant groups identified similar issues, such as the siloed nature of specialties leading to fragmented care [42, 48, 49, 52, 64, 84, 86] and poor care continuity [37, 51, 58, 80], lack of clarity of responsibility [52, 54, 61, 71, 72, 86], insufficient interprofessional communication [35, 60, 66, 71, 78] and a perception that specialists were unwilling to offer medical advice beyond their area of expertise [44, 58, 78]. Limited functionality of electronic health record systems was noted by healthcare professionals to perpetuate these experiences, impacting on the reliability of medication history [37, 52, 83] and, in some instances, positioning people living with MLTC as the source of information for healthcare professionals [37, 46]. These challenges may be particularly acute between mental and physical healthcare services; studies including people living with dementia or psychosis reported a lack of service integration and lack of adaptation of physical health care delivery for people with mental health conditions [58, 61, 87]. Three studies, two conducted with patients and informal caregivers, and one with nurses, concluded that a named individual with responsibility for overseeing care for people living with MLTC would improve care coordination [49, 50, 78], although a barrier to creating and maintaining this role, noted by health care professionals, might be the structure of the funding arrangements and performance measurement for specialties [60]. Four studies (9.8%), three of which were conducted with healthcare professionals, and one with patients, and two of which were in low- and middle-income countries, highlighted under-resourcing in hospital care as a barrier to care coordination [42, 53, 64, 88].

### Knowledge

Eight studies (19.5%) highlighted lack of knowledge and experience of treating other conditions as a contributing factor to the lack of joined-up care in hospital settings. Guidelines for clinical practice were perceived by healthcare professionals to be limited [44, 55, 83], with the research evidence base lacking for older people or people with MLTC as a result of exclusion of these groups from clinical trials [44, 48]. This led one study team to suggest that health care professionals treating people with MLTC do not “have a clear script to follow” [68] (p. 573) and, for another, that treatment recommendations may be more subjective rather than evidence-based due to the paucity of research evidence on MLTC populations [44]. Studies including people with dementia noted the lack of training, confidence, and experience of healthcare professionals to provide care for someone with cognitive impairment [44, 57].

### Time and pacing

Insufficient time in the hospital setting to provide care for people with MLTC was a focal point of 16 studies (39.0%), with people living with particular conditions including HIV and dementia perceived by healthcare professionals as needing longer and more frequent appointments [58, 69, 77].

Time and pacing seemed particularly salient in emergency departments and at the point of discharge. The emphasis on rapid and efficient pathways through emergency care, either to admission or discharge, was not perceived to be congruent with the need for more time to understand the needs of people with MLTC in studies involving healthcare professionals and people living with MLTC [37, 53, 67, 70]. Observational data collected during an ethnographic study suggested this may lead to an over-simplification of chronic conditions and a lack of understanding of the cause of symptoms [36]. There were findings in two studies including healthcare professional participants that older people may be perceived by staff as resource-intensive and time-consuming in the emergency department [51, 53]. Prescribing in emergency care settings was perceived by doctors and pharmacists in two studies to be hurried, creating a focus on prescribing for acute presentations which could lead to long-term conditions being overlooked [48, 83].

Discharge was another point on the care pathway that was perceived as being problematic in terms of time. Discharge was described as being premature or rushed [45, 49, 63, 79], which could leave people living with MLTC and their caregivers feeling ill-prepared to navigate life with multiple long-term conditions after an in-patient stay [38, 45, 49, 65]. Eleven studies (26.8%), conducted in high-income countries, identified the recent trends in health policy towards shortened hospital stays, efficient discharges, and a prioritisation of highly specialised and measurable care for distinct conditions as disadvantageous for the MLTC population.

### Person-centred care

Seventeen studies (41.5%) presented findings on person-centred care for people with MLTC in specialist settings. Despite being a common concept underpinning the body of literature, few authors offered a definition of person-centred care. Exceptions included a study in the US with healthcare professionals caring for people with HIV and HCV [69], which cited Mead and Bower’s (2000) conceptualisation, a study in Pakistan of nurses caring for people living with MLTC which adopted Morgan and Yoder’s (2012) biopsychosocial-spiritual definition [88], and a study conducted in Canada which cited Stewart’s (1995) model [64].

Knowing the person living with MLTC was identified by healthcare professionals as a crucial component of care in several studies [39, 58, 68, 83]. However, in an example of divergence between professional and patient or family perspectives, studies reported that people living with MLTC and informal caregivers perceived a lack of individualised care [41, 50, 51, 64, 77, 81, 84, 88]. Studies involving only people with MLTC reported findings of poor communication with clinicians [63, 64, 70, 86], with staff perceived to prefer to focus on tangible symptoms [70] rather than on the whole person.

Twelve studies (29.3%), seven of which included people living with dementia, presented data relating to decision-making processes in hospital care for people with MLTC. Studies from the perspective of people living with MLTC highlighted the need for greater involvement in decision-making and the opportunity to share the important aspects of their conditions with healthcare professionals [50, 66, 70]. Building a trusting collaborative relationship between people with MLTC, informal caregivers, and staff was perceived to require time and consistency in two studies reporting the perspectives of staff and informal caregivers [69, 74].

### Mental capacity and decision-making

Studies including people with dementia highlighted the challenges that the condition presented for shared decision-making [57, 76], particularly relating to cancer treatment, where concerns were raised over the person with cancer and dementia being unable to fully participate in treatment decision-making, communicate information about treatment side-effects, or recall their surgery. Decision-making led to an increased reliance on family members in studies exploring the perspectives of informal caregivers and healthcare professionals [39, 87] which could cause additional stress [56]. Some studies reported that family members could feel excluded from engaging in decision-making processes [44, 47, 87].

### Support from informal caregivers

Thirteen studies (31.7%) highlighted support from informal caregivers as a key element in the experience of MLTC hospital care, with some studies finding that family members assumed the roles of advocacy and care coordination [56, 62, 73]. In studies including the patient perspective, the absence of an involved family member could reduce access to support [44, 66, 82]. Two studies offering insights into caregiver perspectives suggested that healthcare professionals needed to understand the level of caregiver involvement, assess their ability to provide support to their relative and understand the impact of ageing on caregivers’ abilities to continue to provide support [56, 75]. Some studies illuminated tensions in the provider-informal caregiver relationship; one study of healthcare professionals in Sweden, for example, noted that family members could create ethical dilemmas by asking the clinician not to report illness details to patients [51].

### Broader social context

A small number of studies explored the broader social context of health conditions, highlighting conditions such as HIV and TB which may be normalised in hospital environments but stigmatised in the community [45, 62, 69]. Deep-rooted gender issues were described in the two studies conducted in South Asia, with women’s reliance on men to transport them to appointments [88], and poorer treatment of women with TB and depression [62] influencing access to and experience of hospital care. Socioeconomic status, poor housing, and lack of transport were seen as compounding the challenges associated with accessing services [41, 45, 62, 63], and while gender was consistently reported in these four studies, with evenly balanced samples, other characteristics were less routinely described. All the participants in one study [45] were receiving financial support or government disability services, and, in another [41], were described as low or middle income. Occupation was reported in two studies, with approximately half of the participants not in paid employment [41, 62], and housing status in one [45]. Ethnicity was less well-reported, with the exception of a study on Aboriginal and Torres Strait Islander peoples [63] which identified a need for culturally appropriate MLTC care.

Sociocultural understandings of health conditions may be reflected in clinical perspectives of dementia [44], with some perceptions of negative attitudes or stereotyping behaviour among staff caring for older people [51] or people with mental health conditions [35].

## Discussion

We undertook a scoping review of published qualitative studies on experiences of care for people living with MLTC in hospital care settings to identify the breadth and nature of qualitative literature, the key concepts underpinning the knowledge base, and to highlight gaps for future research. A key finding of our review, which identified 54 papers, underlines the complexity of specialist care provision for people with MLTC, and illuminates the tension between a desire to provide person-centred care that attends to the needs of people with MLTC and a target-driven system of specialist care subject to increasing pressures to accelerate care pathways [89, 90].

Challenges to coordinating care across specialties were the most consistent finding in the review. With the exception of dementia and psychosis, which appeared to present additional challenges to integrating care, we observed little variation in findings across countries or health conditions, although this may require further interrogation in studies where MLTC, and the conditions included, are more clearly defined. Complementing existing evidence which suggests that care coordination is challenging in primary care for people with MLTC [91, 92], this review indicates that the current specialist care provision in hospital settings is also poorly suited to the needs of people living with two or more long-term conditions, and that greater effort needs to be made to integrate services and to deliver care that is responsive to the needs of people with MLTC [16].

Person-centred care formed a key concept within the reviewed studies. However, in common with other health research adopting this concept [93], few authors offered a definition of person-centred care, and there was some variability in those put forward. In general, individualising care, being listened to by healthcare professionals, and being involved in decision-making processes was perceived as important by people living with MLTC and informal caregivers.

Similar to Ho et al.’s systematic review of quantitative studies of multimorbidity [4], we found that MLTC was typically defined as the co-existence of two or more long-term conditions in an individual, and was rarely afforded further clarification. It should be noted that some of these papers were more closely aligned with definitions of comorbidity [94]; however, in wishing to take an inclusive approach, these papers were included. Definitions founded on the number of body systems affected by morbidities (such as complex multimorbidity, defined by Harrison et al. [95] as three or more conditions affecting three or more body systems) were not in evidence in this literature. Moreover, around a quarter of studies in our review did not provide any definition of MLTC. In a field as complex and rapidly expanding as MLTC research, we concur with Ho et al. [4], that, while study populations may appropriately vary according to the research question, achieving greater consistency and transparency in the definition of MLTC will enhance coherence and comparability.

Social and health inequalities were not a core element of this body of literature, yet MLTC are known to be associated with socioeconomic deprivation, with earlier onset of MLTC among people living in socially deprived areas [7, 96] and evidence that minority communities are disproportionately affected by MLTC [97, 98]. While qualitative approaches cannot provide prevalence estimates, they nevertheless present an opportunity to gain insights into the ways in which people’s life circumstances might impinge on capacity to prioritise health and to access and engage with healthcare [99].

Of the five previous systematic or scoping reviews we identified on MLTC care [20–24], none had focussed on the hospital setting. However, all highlighted similar findings to our own, namely poorly coordinated healthcare and challenges delivering person-centred or holistic care. Lack of guidelines for MLTC care was also highlighted in two reviews [20, 23] yet the important role informal caregivers may play was only emphasised in one [21]. Three of the reviews highlighted communication between primary and secondary care as a barrier to care coordination [20, 21, 23], with one in particular noting the potential antagonism between the holistic ethos of general practice and the specialist focus on individual body systems [20]. Time and pacing, a key finding from our review and perhaps more salient in the hospital setting, was not prominent in these other reviews, though limited time for GP consultations was noted as a potential barrier to optimal care [20].

### Gaps in the knowledge base

The studies identified in this review have employed a range of qualitative approaches to provide insights into the experiences of hospital care for people living with MLTC from the perspectives of people receiving care, informal caregivers and healthcare providers. We can identify at least six clear gaps in the evidence base. Firstly, most studies were cross-sectional, capturing snapshots of experiences of hospital care rather than the experience of receiving or delivering hospital care over time. Studies that had a longitudinal design were either ethnographies [37, 41, 52, 55] or collected data through repeated interviews. Of the latter, most data collection was conducted over a period of a few weeks [45, 70, 73], with one exception where patients were followed up for between 5 and 9 months [75]. Consequently the studies were limited in the extent to which they could generate understandings of interactions with hospital care systems over time and how these might be shaped by biographical influences.

Secondly, the studies tended to focus on older populations, with only a small number of studies recruiting people living with MLTC below the age of 40 [45, 62–64, 76]. While ageing is associated with higher risk of MLTC, a study in Scotland found that more than half of people with MLTC were younger than 65 years of age, and that this was socially patterned, with socioeconomic deprivation associated with younger age at onset of MLTC [7, 96]. Further evidence suggests that MLTC are associated with ethnicity [100, 101] and gender [102] yet with the notable exception of the Richmond Group of Charities Taskforce study on MLTC and health equity [99], how sociodemographic characteristics may intersect to structure the experience of MLTC remains under-researched, and is not explored in the reviewed studies. Insights into this could be gained through life course approaches which can investigate ageing with MLTC and engaging with hospital care over time.

Thirdly, although the review identified several studies on dementia co-existing with other conditions, there was little focus on other types of mental health condition such as severe mental illness or common mental health disorders, despite the fact that associations between, for example, severe mental illness and diabetes [103], and depression and comorbid long-term physical health conditions are well-established [104].

Fourthly, the importance of informal support for people with MLTC receiving hospital care was highlighted in around a third of studies, with informal caregivers assisting with care coordination, managing multiple appointments and medications, and having a role in decision-making, particularly when the person receiving care was living with dementia. However, studies focussed on the experiences of a single person providing support, which rests on the assumption that only one person undertakes a caregiving role, and neglects broader supportive networks that some people with MLTC may have. Only a few studies [44, 66] articulated experiences of people with MLTC who did not have informal support. The limited evidence available suggests that lack of such support could affect access to services, which warrants further investigation.

Fifthly, with the exception of a study on cancer and dementia [32] which found that the hospital environment was not suited for people living with dementia, studies did not offer findings on the environment of the hospital as a physical institution in which care delivery took place. Experiences of navigating the hospital landscape with MLTC, which are associated with functional impairment [105] remain underexplored. In the context of greater centralisation of hospital services [106], and overcrowding in emergency departments leading to care being undertaken in corridors [107], there is an opportunity for future studies to illuminate the experience of the physical environment in which care takes place.

Finally, power dynamics of clinician-patient interactions in clinical spaces were only explored in depth in one study [47], and, while findings from the body of literature could be related to care quality, only two studies explicitly focussed on the concept of quality of care for MLTC [70, 84].

### Potential future directions

Table 6.

**Table 6:** Overview of evidence gaps and potential future directions for qualitative research on MLTC hospital care

Gap in the current evidence base	Potential approach to addressing this gap in future qualitative research
How intersecting inequalities may shape MLTC experiences and interactions with hospital care in the context of lived lives	Life course /biographical narrative
Experiences of younger people living with MLTC	Sampling younger people with MLTC
Experiences of people living with mental health conditions	Sampling people with severe mental illness and common mental disorders
Diversity in access to informal care	Sampling people with no informal support or > 1 informal caregiver and comparing and contrasting their experiences
Navigation of hospital built environments	Human geography
Dynamics of clinician-patient interactions	Medical sociology Discourse analysis
Quality of care	Explicit focus on concept of care quality

### Strengths and limitations

Our review was novel in aiming to identify and describe the findings from qualitative research on the experiences of hospital care for people with MLTC. We followed established methods for scoping reviews [25, 26], including a systematic electronic search strategy supplemented with citation tracking and, as a result, were able to identify and summarise studies from 14 countries.

We recognise three main limitations of our review. First, we chose to focus on peer-reviewed literature published after 2010 to identify research reflecting experiences most likely to be relevant to the current context of hospital care, and we did not conduct searches of grey literature to ensure that the task of reviewing titles and abstracts was manageable in scale. However, we recognise that some relevant research, published earlier, or not published in peer-reviewed journals, may therefore have been missed. Additionally, although we used systematic searching methods and citation tracking, we did not contact authors or hand-search journals. Second, complexities around the definition and operationalisation of MLTC, and the frequent conflation of MLTC with age, meant that we were presented with a decision on whether to include a small number of studies that purported to study MLTC but recruited participants solely on the inclusion criterion of older age. We opted to exclude these studies as we could not be certain that the participants had MLTC and were reluctant to perpetuate notions of MLTC as an inevitable aspect of older age. Additionally, if we had wished to capture all studies of this nature, we would have had to expand our search terms to include all studies of older adults regardless of reported MLTC status. Third, in wishing to illuminate in-depth experiences, we focused our review on qualitative studies. We acknowledge that further valuable insights could be gained from quantitative surveys of the views and experiences of people living with MLTC, informal caregivers and healthcare professionals.

## Conclusions

The accumulating evidence of the current and anticipated scale of MLTC, and its impact on quality of life and demand for healthcare, have led to calls to prioritise MLTC research. This review of qualitative studies has illuminated tensions between a drive to provide individualised person-centred care for people with MLTC in hospital settings, and a system which is moving towards greater clinical specialism and accelerated care pathways. More integrated models of care may enable the needs of people living with multiple long-term conditions to be better met in the hospital setting.

### Supplementary Information

**Additional file 1.** Study protocol.

**Additional file 2.** Electronic database search strategies.

**Additional file 3.** Data extraction template.

## Data Availability

No data were generated for this study. The study protocol, search strategies and data extraction template are available as Additional files.

## Abbreviation

MLTC: Multiple long-term conditions
