# Supplementary material for: Experiences of hospital care for people with multiple long-term conditions: a scoping review of qualitative research
Source: BMC Med. 2024 Jan 17;22:25. doi: 10.1186/s12916-023-03220-y (PMC10792930; doi:10.1186/s12916-023-03220-y)
Supplement: Supplementary file 1 — Additional file 1. Study protocol. [file 12916_2023_3220_MOESM1_ESM.docx]

**Experiences of hospital care for people with multiple long-term conditions: a scoping review of qualitative research. S Bellass et al.**

**ADDITIONAL FILE 1: Review Protocol**

**Headings are 36**

**Experiences of UK hospital care for multiple long-term conditions: scoping review protocol**

Sue Bellass, Rachel Cooper, Tom Scharf 29.07.2022

**Title:** Experiences of UK hospital care for multiple long-term conditions: scoping review protocol

**Review team**

Dr Sue Bellass, Linda Errington, Prof Rachel Cooper, Prof Tom Scharf, Dr Adam Runacres, Dr Kelly Bowden Davies, Dr Jodi Ventre, Prof Miles Witham, Prof Sian Robinson, Prof Avan Sayer. Posthumous recognition: Dr Richard Dodds

**Timeline**

Mar 2022 – Dec 2022

**Aim**

The aim of this scoping review is to identify and synthesise qualitative evidence on the experiences of hospital care for people living with multiple long-term conditions. This evidence may be from the perspectives of patients, family members or friends who provide support, or professionals involved in care delivery. Scoping this literature will identify key concepts and gaps in the evidence base which will inform empirical qualitative studies to be undertaken as part of ADMISSION work packages 3 and 4.

**Approach**

The scoping review framework devised by Arksey and O’Malley (2005) will be used to structure this review. The five core elements in the framework are as follows:

1. Identifying the research question

2. Identifying relevant studies

3. Study selection

4. Charting the data

5. Collating, summarising and reporting the results

Further detail on each stage is presented below.

1. **Identifying the research question**

*Research question framework*

Concerns about the applicability of the concepts incorporated within the PICO question framework (Population-Intervention-Comparison-Outcome) for the retrieval of qualitative health research literature led to the development of the SPIDER tool (Sample-Phenomenon of Interest-Design-Evaluation-Research Type) (Cooke et al. 2012). However, scoping reviews require relatively sensitive search strategies, and Methley et al. (2014), in their comparative study of research question frameworks for qualitative syntheses, found that use of SPIDER produced searches with lower sensitivity than PICO.

For this review, the PICo framework (Population-Phenomenon of Interest-Context) has been selected which is constituted by concepts more relevant to qualitative research (Stern et al., 2014), yet, with fewer criteria than SPIDER, may have greater sensitivity. Inclusion of Study Type targets literature with empirical relevance to the research question.

**Table 1: PICo Framework**

| P | Population | People with experience of multiple long-term conditions (MLTC; including both physical and mental health conditions) as patients, family members and friends who provide support, or staff delivering care |
| --- | --- | --- |
| I | Phenomenon of Interest | Experience |
| Co | Context | NHS secondary care |
| S | Study type | Qualitative |

*Research question:* What are the experiences of UK hospital care for MLTC of patients, family members and friends who provide support (informal caregivers) and care professionals?

In accordance with the threefold aims of scoping reviews, to identify the nature and range of evidence, the key concepts underpinning the literature and the gaps in the literature, the scoping review questions are as follows:

*Scoping review questions:*

1. What is the nature, range and extent of published qualitative literature exploring hospital care experiences of people living with MLTC, informal caregivers and healthcare professionals?
2. What experiences of hospital care have been reported in the literature?
3. What gaps exist in the knowledge base that might be addressed by future research?
4. **Identifying relevant studies**

This review will identify qualitative research studies published in peer-reviewed journals and grey literature. The following databases will be used: MEDLINE, CINAHL, PsycInfo, Proquest Social Science Premium, Web of Science, Scopus and Embase.

Scoping reviews require a sensitive and systematic searching strategy to identify and map relevant literature, ensuring that key concepts and gaps in the knowledge base can be confidently identified. However, highly sensitive strategies return considerable amounts of ineligible literature. During preliminary all-text searches, for instance, the search term ‘hospital’ identified author affiliations on numerous ineligible papers (in addition to relevant articles). Therefore, to increase the specificity of the strategy, electronic searches will be restricted to title, abstract and keyword fields. Supplementary searches, detailed below, will be implemented to minimise the risk of overlooking potentially relevant literature.

**Table 2: Sample search string (MEDLINE via Ovid)**

| **Concept** | **Search string** | **Adapted from** |
| --- | --- | --- |
| Multimorbidity | 1. exp Comorbidity/ 2. (co?morbidit* or multi?morbidit* or multiple LTC* or poly?morbidit* or multi?condition* or multiple chronic* or morbidity burden or multiple health problem*).ti,ab,kf. 3. ((multiple or coexisting or co-existing or concurrent or con-current or comorbid or co-morbid) adj2 (disease* or illness* or condition* or diagnos* or morbid*)).ti,ab,kf. 4. 1 or 2 or 3 | Ho, I.S. et al. (2021) |
| Hospital | 1. (hospital* or acute setting* or acute care or inpatient* or ward* or secondary care or tertiary care or specialist care).ti,ab,kf. 2. exp Hospitals/ 3. exp Hospitalization/ 4. Inpatients/ 5. Secondary Care/ 6. Tertiary Care/ 7. or/5-10 | Burton, J. K. et al. (2021) |
| Qualitative | 1. (qualitative or mixed metho* or interview* or grounded theory or ethnograph* or phenomenol* or focus group* or interpretive phenomenological analysis or IPA or narrative or thematic analysis).ti,ab,kf. 2. exp qualitative research/ 3. 12 or 13 | Kvarnström et al. (2021) |
| Combined search | 1. 4 and 11 and 14 |  |
| Limiters | 16.     limit 15 to (english language and yr="2010 -Current") |  |

*Supplementary searches*

Supplementary searches will be conducted including citation tracking, and contacting authors where appropriate.

*Documenting searches*

Records will be made of all literature searching activities, guided by the PRISMA-S checklist (Rethlefsen et al., 2021), to ensure reproducibility of the search strategy. The PRISMA-S checklist will be made available as a supplementary document.

1. **Study selection**

Given our empirical interest in NHS care delivery, we aim to only include studies that report evidence relating to the UK context in the review. However, studies conducted in other countries, but that meet all other criteria, will not be excluded until the full-text stage of the review. This allows for the possibility of extending the review to include studies relating to international secondary care systems in the event that there is insufficient data from UK-only studies.

As hospital care delivery systems are subject to regular restructuring, only studies published since 2010 will be eligible for inclusion. Consistent with scoping review methodology, studies will not be excluded on the basis of poor quality. While systematic, scoping and narrative review studies will be excluded at the title and abstract screening stage, relevant reviews will be later catalogued in the reference management software (Endnote) to enable examination of reference lists to identify potentially eligible studies.

**Table 3: Eligibility criteria**

| **Inclusion criteria** | **Exclusion criteria** |
| --- | --- |
| Qualitative studies (or mixed methods studies with a qualitative component) that report evidence relating to the UK context on MLTC care in hospital | Quantitative research studies |
| Studies published from 1^st^ Jan 2010 | Studies that do not include evidence relating to the UK context |
| English language | Study protocols or literature reviews |

*Screening*

Sources will be independently screened using Covidence, a software platform designed to support systematic reviews, by a team of researchers (SB, RC, LE, AR, KBD, JV). Any disagreements on eligibility will be resolved in consultation with senior academics (RC,TS).

A PRISMA flow diagram (automatically created on Covidence) will provide a visual representation of the study selection process, including reasons for exclusion.

1. **Charting the data**

Data will be extracted from included studies into a data charting table for ease of comparison. At a minimum the data charting table will include: author(s), year of publication, location/ setting of study, definition of multiple long-term conditions and health conditions studied, study aim and design, sample characteristics, key findings, reference to health inequalities, limitations, identified areas for future research and conceptual/ theoretical framework (if applicable).

1. **Collating, summarising and reporting**

The findings from the scoping review will be summarised according to key themes identified within the literature. A narrative will be created which will articulate the contours of the knowledge base, and identify gaps and areas for future research.

*Reporting*

The PRISMA-ScR (Tricco et al. 2018), a reporting checklist for scoping reviews, will be completed as a supplementary document to enhance the transparency of the review process.
