# Supplementary material for: Experiences of hospital care for people with multiple long-term conditions: a scoping review of qualitative research
Source: BMC Med. 2024 Jan 17;22:25. doi: 10.1186/s12916-023-03220-y (PMC10792930; doi:10.1186/s12916-023-03220-y)
Supplement: Supplementary file 2 — Additional file 2. Electronic database search strategies. [file 12916_2023_3220_MOESM2_ESM.docx]

**Experiences of hospital care for people with multiple long-term conditions: a scoping review of qualitative research. S Bellass et al.**

**ADDITIONAL FILE 2 : Search strategies employed in different databases**

**MEDLINE (Ovid)**

**(n=2206)**

1. exp Comorbidity/
2. (co?morbidit* or multi?morbidit* or multiple LTC* or poly?morbidit* or multi?condition* or multiple chronic* or morbidity burden or multiple health problem*).ti,ab,kf.
3. ((multiple or coexisting or co-existing or concurrent or con-current or comorbid or co-morbid) adj2 (disease* or illness* or condition* or diagnos* or morbid*)).ti,ab,kf.
4. 1 or 2 or 3
5. (hospital* or acute setting* or acute care or inpatient* or ward* or secondary care or tertiary care or specialist care).ti,ab,kf.
6. exp Hospitals/
7. exp Hospitalization/
8. Inpatients/
9. Secondary Care/
10. Tertiary Care/
11. or/5-10
12. (qualitative or mixed metho* or interview* or grounded theory or ethnograph* or phenomenol* or focus group* or interpretive phenomenological analysis or IPA or narrative or thematic analysis).ti,ab,kf.
13. exp qualitative research/
14. 12 or 13
15. 4 and 11 and 14
16. limit 15 to (english language and yr="2010 -Current")

**Embase** **(Ovid)**

**(n=6193)**

1. Comorbidity/
2. (co?morbidit* or multi?morbidit* or multiple LTC* or poly?morbidit* or multi?condition* or multiple chronic* or morbidity burden or multiple health problem*).ti,ab,kf.
3. ((multiple or coexisting or co-existing or concurrent or con-current or comorbid or co-morbid) adj2 (disease* or illness* or condition* or diagnos* or morbid*)).ti,ab,kf.
4. 1 or 2 or 3
5. (hospital* or acute setting* or acute care or inpatient* or ward* or secondary care or tertiary care or specialist care).ti,ab,kf.
6. exp Hospital/
7. exp Hospitalization/
8. exp hospital patient/
9. Secondary Health Care/
10. Tertiary Health Care/
11. Emergency Care/
12. or/5-11
13. (qualitative or mixed metho* or interview* or grounded theory or ethnograph* or phenomenol* or focus group* or interpretive phenomenological analysis or IPA or narrative or thematic analysis).ti,ab,kf.
14. exp qualitative research/
15. 13 or 14
16. 4 and 12 and 15
17. limit 16 to (english language and yr="2010 -Current")

**PSYCINFO (Ovid)**

**(n=798)**

1. Comorbidity/
2. (co?morbidit* or multi?morbidit* or multiple LTC* or poly?morbidit* or multi?condition* or multiple chronic* or morbidity burden or multiple health problem*).ti,ab,id.
3. ((multiple or coexisting or co-existing or concurrent or con-current or comorbid or co-morbid) adj2 (disease* or illness* or condition* or diagnos* or morbid*)).ti,ab,id.
4. 1 or 2 or 3
5. (hospital* or acute setting* or acute care or inpatient* or ward* or secondary care or tertiary care or specialist care).ti,ab,id.
6. exp Hospitals/
7. exp Hospitalization/
8. Hospitalized Patients/
9. or/5-8
10. (qualitative or mixed metho* or interview* or grounded theory or ethnograph* or phenomenol* or focus group* or interpretive phenomenological analysis or IPA or narrative or thematic analysis).ti,ab,id.
11. exp qualitative methods/
12. 10 or 11
13. 4 and 9 and 12
14. limit 13 to (english language and yr="2010 -Current")

**SCOPUS (Elsevier)**

**(n=1059)**

## ( ( TITLE-ABS-KEY ( co?morbidit*  OR  multi?morbidit*  OR  multiple  AND ltc*  OR  poly?morbidit*  OR  multi?condition*  OR  "multiple chronic*"  OR  "morbidity burden"  OR  "multiple health problem*" ) )  OR  ( TITLE-ABS-KEY ( ( multiple  OR  coexisting  OR  co-existing  OR  concurrent  OR  con-current  OR  comorbid  OR  co-morbid )  W/2  ( disease*  OR  illness*  OR  condition*  OR  diagnos*  OR  morbid* ) ) ) )  AND  ( TITLE-ABS-KEY ( hospital*  OR  "acute setting*"  OR  "acute care"  OR  inpatient*  OR  ward*  OR  "secondary care"  OR  "tertiary care"  OR  "specialist care" ) )  AND  ( TITLE-ABS-KEY ( qualitative  OR  "mixed metho*"  OR  interview*  OR  "grounded theory"  OR  ethnograph*  OR  phenomenol*  OR  "focus group*"  OR  "interpretive phenomenological analysis"  OR  ipa  OR  "narrative or thematic analysis" ) ) AND ( LIMIT-TO ( PUBYEAR , 2010-2022 ) AND ( LIMIT-TO ( PUBYEAR , 2010-2022 )   (LIMIT TO ( LANGUAGE, "English"))

**WEB OF SCIENCE (Clarivate)**

**(n=879)**

Limited to English language 2010-2022

1. (multiple OR coexisting OR co-existing OR concurrent OR con-current OR comorbid OR co-morbid) NEAR/2 (disease* OR illness* OR condition* OR diagnos* OR morbid*) (Topic)

2. co?morbidit* OR multi?morbidit* OR “multiple ltc*” OR poly?morbidit* OR multi?condition* OR “multiple chronic*” OR “morbidity burden” OR “multiple health problem*” (Topic)

3. #1 OR #2

4. qualitative OR “mixed metho*” OR interview* OR “grounded theory” OR ethnograph* OR phenomenol* OR “focus group*” OR “interpretive phenomenological analysis” OR ipa OR “narrative or thematic analysis” (Topic)

5. hospital* OR “acute setting*” OR “acute care” OR inpatient* OR ward* OR “secondary care” OR “tertiary care” OR “specialist care” (All Fields)

6. #3 AND #4 AND #5

**CINAHL (Ebsco)**

**(n=1035)**

S1= TX co?morbidit*  OR  multi?morbidit*  OR  "multiple ltc*"  OR  poly?morbidit*  OR  multi?condition*  OR  "multiple chronic*"  OR  "morbidity burden"  OR  "multiple health problem*"

S2= TX ( multiple  OR  coexisting  OR  co-existing  OR  concurrent  OR  con-current  OR  comorbid  OR  co-morbid )  N2  ( disease*  OR  illness*  OR  condition*  OR  diagnos*  OR  morbid* )

S3= S1 OR S2

S4= TX hospital* or "acute setting*" or "acute care" or inpatient* or ward* or "secondary care" or "tertiary care" or "specialist care"

S5= TX qualitative or "mixed metho*" or interview* or "grounded theory" or ethnograph* or phenomenol* or "focus group*" or "interpretive phenomenological analysis" or IPA or narrative or "thematic analysis"

**SOCIAL SCIENCE PREMIUM (Proquest)**

**(n= 47)**

su(hospital* OR acute setting* OR acute care OR inpatient* OR ward* OR "secondary care" OR "tertiary care" OR "specialist care") AND (su(co?morbidit* OR multi?morbidit* OR "multiple LTC*" OR poly?morbidit* OR multi?condition* OR "multiple chronic*" OR "morbidity burden" OR "multiple health problem*") OR su((multiple OR coexisting OR co-existing OR concurrent OR con-current OR comorbid OR co-morbid) NEAR/2 (disease* OR illness* OR condition* OR diagnos* OR morbid*))) AND su(qualitative OR "mixed metho*" OR interview* OR "grounded theory" OR ethnograph* OR phenomenol* OR "focus group*" OR "interpretive phenomenological analysis" OR IPA OR narrative OR "thematic analysis") 2010-2022 English Language
